# Identification and Clinical Validation of a Novel 4 Gene-Signature with Prognostic Utility in Colorectal Cancer

**DOI:** 10.3390/ijms20153818

**Published:** 2019-08-05

**Authors:** Pankaj Ahluwalia, Ashis K. Mondal, Chance Bloomer, Sadanand Fulzele, Kimya Jones, Sudha Ananth, Gagandeep K. Gahlay, Saleh Heneidi, Amyn M. Rojiani, Vamsi Kota, Ravindra Kolhe

**Affiliations:** 1 Department of Pathology, Anatomic Pathology Section, Medical College of Georgia at Augusta University, Augusta, GA 30912, USA; 2 Department of Molecular Biology and Biochemistry, Guru Nanak Dev University, Amritsar 143005, India; 3 Department of Orthopedics, Medical College of Georgia at Augusta University, Augusta, GA 30912, USA; 4 Department of Medicine, Hematology Oncology Section, Medical College of Georgia at Augusta University, Augusta, GA 30912, USA

**Keywords:** prognostic, biomarker, tumor, colorectal cancer, gene expression, survival analysis

## Abstract

Colorectal cancer (CRC) is a high burden disease with several genes involved in tumor progression. The aim of the present study was to identify, generate and clinically validate a novel gene signature to improve prediction of overall survival (OS) to effectively manage colorectal cancer. We explored The Cancer Genome Atlas (TCGA), COAD and READ datasets (597 samples) from The Protein Atlas (TPA) database to extract a total of 595 candidate genes. In parallel, we identified 29 genes with perturbations in > 6 cancers which are also affected in CRC. These genes were entered in cBioportal to generate a 17 gene panel with highest perturbations. For clinical validation, this gene panel was tested on the FFPE tissues of colorectal cancer patients (88 patients) using Nanostring analysis. Using multivariate analysis, a high prognostic score (composite 4 gene signature—*DPP7/2*, *YWHAB*, *MCM4* and *FBXO46*) was found to be a significant predictor of poor prognosis in CRC patients (HR: 3.42, 95% CI: 1.71–7.94, *p* < 0.001 *) along with stage (HR: 4.56, 95% CI: 1.35–19.15, *p* = 0.01 *). The Kaplan-Meier analysis also segregated patients on the basis of prognostic score (log-rank test, *p* = 0.001 *). The external validation using GEO dataset (GSE38832, 122 patients) corroborated the prognostic score (HR: 2.7, 95% CI: 1.99–3.73, *p* < 0.001 *). Additionally, higher score was able to differentiate stage II and III patients (130 patients) on the basis of OS (HR: 2.5, 95% CI: 1.78–3.63, *p* < 0.001 *). Overall, our results identify a novel 4 gene prognostic signature that has clinical utility in colorectal cancer.

## 1. Introduction

Colorectal cancer (CRC) affects nearly 1.4 million individuals every year, which makes up to 10% of the global burden of cancer [1]. According to 2019 cancer statistics report, colorectal cancer caused third highest number of deaths due to cancer in United States [2]. The progress in early detection, surgical and chemotherapeutic interventions have significantly reduced the mortality rate, however, the high relapse and variable survival among the patients highlights the need of better prognostic biomarkers [3]. Several recent studies have identified gene expression signatures in cancer that have prognostic utility [4,5,6]. OncotypeDX [7], GeneFx Colon [8] Coloprint [9] signatures are available and are currently being evaluated independently in multiple independent cohorts [10]. There is need for new signatures as all the existing prognostic signatures have been shown to offer only a marginal clinical utility compared to conventional risk factors [11]. Further, a robust risk-gene signature is required to further assist clinicians to tailor personalized treatment for diversity of CRC patients. Over the past few years, several consortium efforts have yielded massive data on multiple types of cancers. The TCGA Research network is one such project with 2.5 petabytes of data that catalogs DNA sequences and its modifications along with transcriptome data of more than 11,000 individuals in over 30 types of cancers [12]. Building on TCGA datasets, secondary databases like TPA and cBioportal can provide hundreds of potential prognostic genes. These genes need additional validation through independent studies and our study is one such effort.

The protein atlas (TPA) database has analyzed transcriptome variation with respect to clinical outcome in 17 major cancers [13]. Another platform, the cBioportal is a graphic web interface to explore aberrations at the genetic, epigenetic and expressional level in multiple types of cancer [14]. The top hits from TPA database and cBioportal were combined to build a prognostic gene panel. The resulting 17-gene panel was internally tested on Formalin fixed, paraffin embedded (FFPE) tissues of CRC patients. In the past, FFPE tissues with clinical information have been instrumental in facilitating prognostic biomarker discovery [15,16]. Additionally, RNA molecules identified in FFPE tumor tissues have been shown to be of the same high quality as that seen in fresh frozen tissue [17]. Clinically, the overall survival analysis based on mRNA expression, has also shown consistent results between fresh frozen and FFPE tissues [18,19]. In an effort to explore differential expression between normal and tumor tissues, GEPIA (Gene Expression Profiling Interactive Analysis) database was accessed. GEPIA collates normal gene expression from normal TCGA database and GTEx Genotype-Tissue Expression (GTEx) project [20,21]. The aim of this study was to identify clinically actionable candidate genes from both TPA database and cBioportal and then to validate those genes internally and externally using FFPE tissues from CRC patient and independent GEO (Gene Expression Omnibus) datasets.

## 2. Results

### 2.1. Exploratory Analysis to Build 17-Gene Panel

To identify risk genes in CRC, 595 candidate genes were accessed through TCGA database through The Human Protein Atlas. The analysis of mRNA expression *z*-score at a threshold of ± 2.0 revealed significant association (*p* < 0.05) of combined gene signature using KM analysis in cBioportal. Among 222 CRC patients, a total of 7 genes in combination showed significant alterations with *PI4K2B* exhibiting the most differential expression in 10% of patients (Table S1). In parallel, 29 genes with prognostic significance in 6 or more varied types of cancers were also run in cBioportal. Most altered gene expression was observed for 10 genes in COAD dataset with *YWHAB* exhibiting significant changes in 39.10% of CRC patients (Table S2). These 10 genes showed significant prognostic value in > 6 cancers (Table S3). The genes included in the panel are shown in Table 1 along with the comparison between tumor and normal colon gene expression.

### 2.2. Clinicopathological Characteristics of CRC Patients

The clinicopathological features of the patients included in this study are in Table 2. The clinic-pathological parameters included were: age, gender, stage, grade, metastasis, ethnicity, vital status, and chemotherapy, family history of cancer, alcohol and tobacco consumption. The cut-off for age was determined as 68 years which is average age of diagnosis of colorectal cancer. The median survival time of the patients in the low survival and high survival group was 11.8 and 54.1 months respectively. The Pearson’s chi-square test was utilized to analyze the association between expression of individual genes and clincopathological characteristics (Table S4). *CHEK1* showed association with family history of cancer (Pearson χ^2^ test, *p* = 0.02 *). The expression of *LRRC59* was found to be higher in Stage III and Stage IV patients (Pearson χ^2^ test, *p* = 0.01 *). There were no significant associations found for other genes with respect to grade or stage.

### 2.3. Univariate, Multivariate Analysis and Generation of Prognostic Score

In univariate Cox regression analysis, *MCM4* (HR 2.69, *p* = 0.01 *), *YWHAB* (HR 3.76, *p* = 0.001 *), *LRRC59* (HR 2.32, *p* = 0.02 *) and *DPP7/2* (HR 0.38, *p* = 0.02 *) showed significant association with overall survival (Table 3). All combinations were tested that yielded a 4 gene composite signature (*YWHAB*, *MCM4*, *DPP7/2* and *FBXO46*) which showed significant association with overall survival independent of other prognostic factors (HR 5.39, 95% CI: 2.19–15.26, *p* < 0.001 *) (Table 4). Further, independent of other variables, the stage was also found to be significantly associated with OS (HR 2.9, 95% CI: 1.39–6.36, *p* < 0.001 *) (Table 4). Upon multivariate analysis using Cox regression with other clinicopathological features, the resulting associations with overall survival were: prognostic score (HR 3.42, 95% CI: 1.71–7.94, *p* <0.001 *), Age (HR 1.05, 95% CI: 0.35–3.19, *p* = 0.92), Gender (HR 2.34, 95% CI: 0.62–9.37, *p* = 0.20), patient stage (HR 4.56, 95% CI: 1.33–19.15, *p* = 0.01 *), Grade (HR 0.19, 95% CI: 0.02–1.18, *p* = 0.07), ethnicity (HR 2.89, 95% CI: 0.69–12.47, *p* = 0.14), alcohol consumption (HR 7.38, 95% CI: 1.58–38.14, *p* = 0.01 *) and tobacco smoking (HR 0.08, 95% CI: 0.01–0.31, *p* = 0.01 *) (Table 5). The multivariate analysis using only 5 variables (prognostic score, age, stage, ethnicity and alcohol consumption) revealed significant associations between prognostic score (HR 2.6, 95% CI: 1.44–5.10, *p* < 0.001 *), stage (HR 3.24, 95% CI: 1.32–8.63, *p* = 0.009 *) and ethnicity (HR 2.46, 95% CI: 0.92–6.75, *p* = 0.0012 *) (Table S5).

### 2.4. Kaplan-Meier Analysis

**Figure 1:**
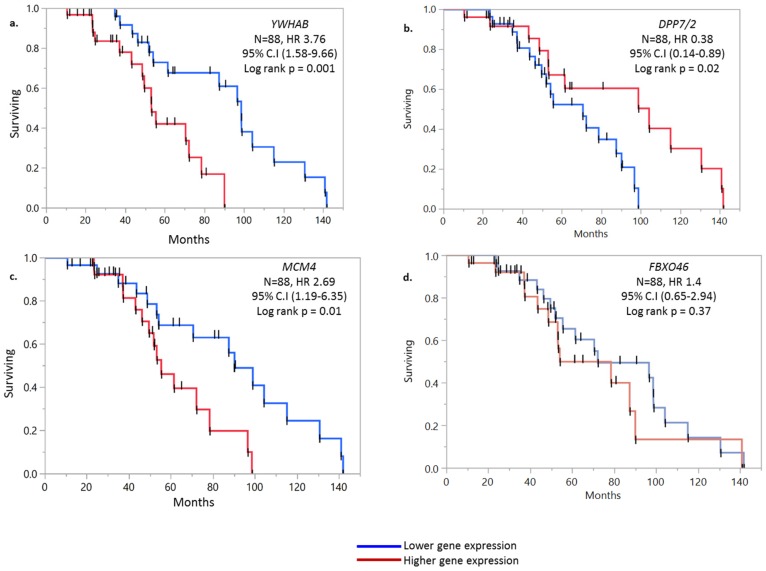
Kaplan-Meier curve of (**a**) *YWHAB*, (**b**) *DPP7/2*, (**c**) *MCM4*, (**d**) *FBXO46* from clinical dataset that were included in generation of prognostic score based on Cox proportion hazard model. The patients were divided into 2 groups, higher and lower based on median gene expression as a cut-off point.

**Figure 2:**
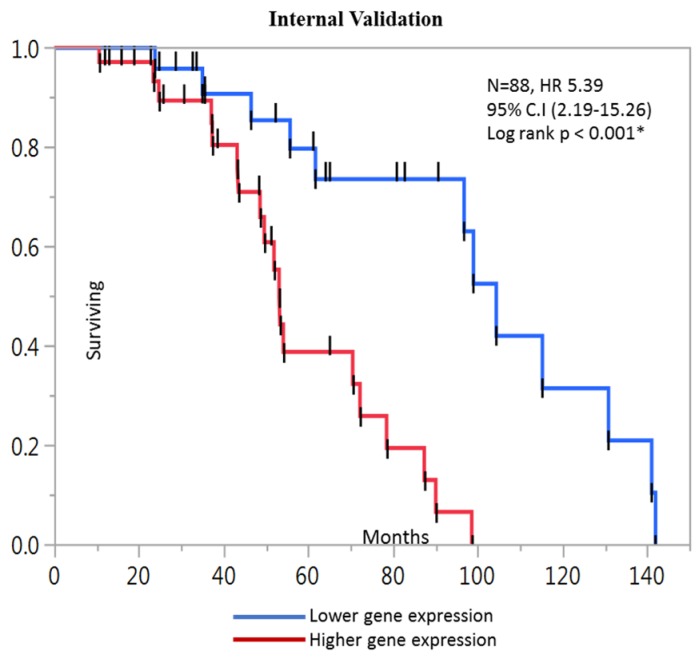
The composite prognostic score differentiated CRC patients (*n* = 88) based on OS. The patients with higher score had poor prognosis compared to lower ones.

Using Kaplan-Meier analyses, we differentiated high-risk group from low-risk based on gene expression (log-rank test, *p* < 0.05). The 4 genes relevant to the prognostic score were ran for KM analysis for both internal and external cohorts. In internal dataset the prognostic significance of 4 genes was: *YWHAB* (HR 3.76, 95% CI: 1.58–9.66, *p* = 0.001), *DPP7/2* (HR 0.38, 95% CI: 0.14–0.89, *p* = 0.02), *MCM4* (HR 2.69, 95% CI: 1.19–6.35, *p* = 0.01) and *FBXO46* (HR 1.4, 95% CI: 0.65–2.94, *p* = 0.37) (Figure 1). The prognostic score generated after summation of regression coefficient and expression value of four genes separated lower and higher survival among groups, with median survival time of 58 vs. 99 months, respectively (log-rank test, *p* < 0.001 *) (Figure 2).

### 2.5. External Validation of Prognostic Score with GEO Microarray Dataset and ROC analysis

**Figure 3:**
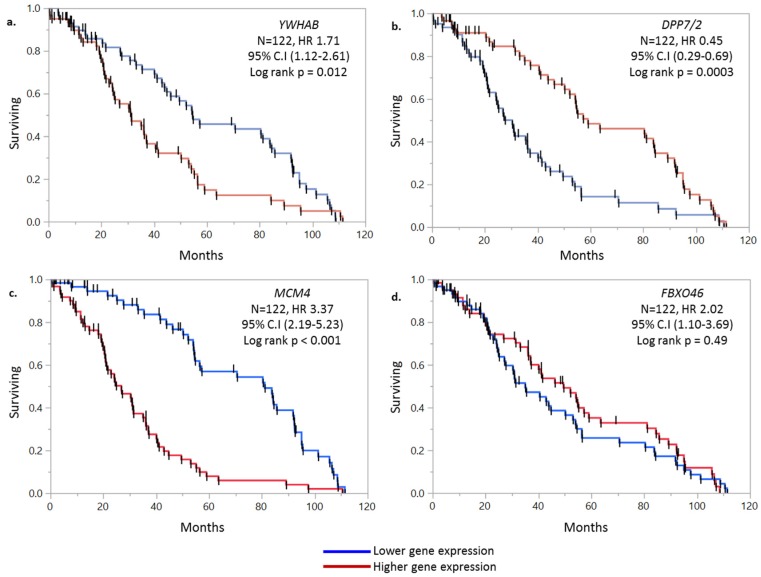
Kaplan-Meier curve of (**a**) *YWHAB*, (**b**) *DPP7/2*, (**c**) *MCM4*, (**d**) *FBXO46* from the external dataset. The median gene expression was used as a cut-off point for higher and lower gene expression groups.

**Figure 4:**
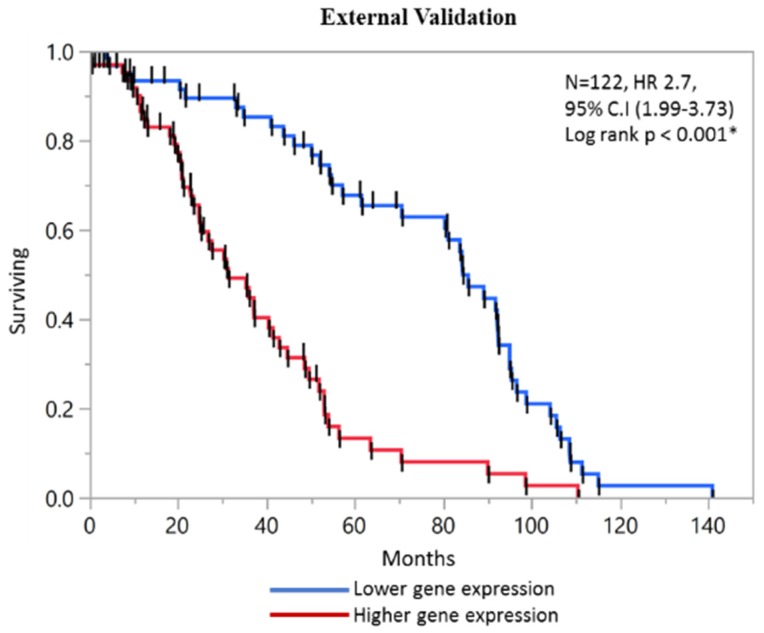
The external validation using independent dataset validated the four gene prognostic score.

To investigate the predictive potential of our four-gene model, an independent GEO microarray dataset (GSE38832) was acquired. The univariate and multivariate cox regression analysis of this dataset is presented in Table S6. The KM analysis of individual gene is presented in Figure 3. In external dataset the prognostic significance of individual genes was: *YWHAB* (HR 1.71, 95% CI: 1.12–2.61, *p* = 0.012), *DPP7/2* (HR 0.45, 95% CI: 0.29–0.69, *p* = 0.0003), *MCM4* (HR 3.37, 95% CI: 2.19–5.23, *p* < 0.001) and *FBXO46* (HR 2.02, 95% CI: 1.10–3.69, *p* = 0.49). The composite prognostic score of all the four genes maintained high significance in achieving separation of lower and high surviving groups, with median of 31 vs. 69 months, respectively (HR 2.7, 95% CI: 1.99–3.73, *p* < 0.001 *) (Figure 4).

Additionally, ROC analysis was performed on the gene signature. In external dataset, The AUC value of survival at less than 1 year, less than 3 years and more than 3 years was found to be 0.529, 0.705 and 0.722 respectively. In Internal dataset, The AUC value of survival at >1 year, <3 year and >3 years is 0.590, 0.534 and 0.607 respectively (Figure S1).

### 2.6. Validation of Prognostic Score in Combined Stage II and Stage III Patients

**Figure 5:**
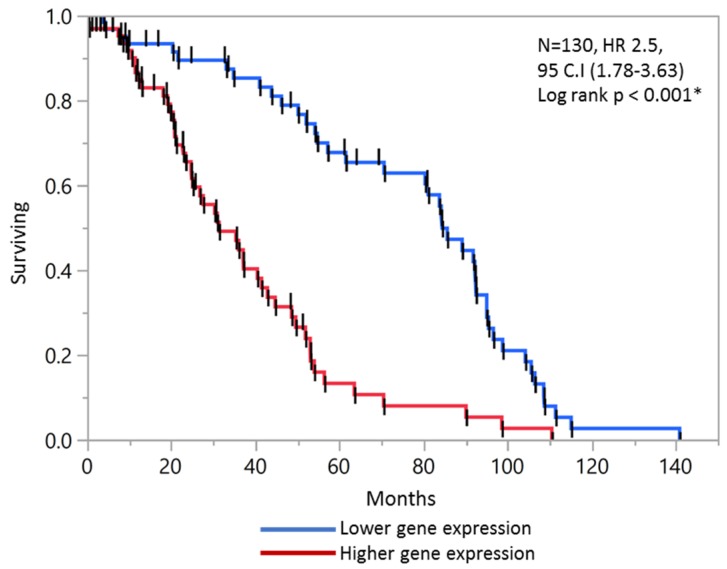
The prognostic score differentiated CRC patients in stage II + III in combined internal and external datasets.

The combined analysis of stage II and stage III patients maintained the prognostic validity of the score. High score was found to be significant predictor of OS (HR 2.5, 95 CI: 1.78–3.63, *p* = 0.001 *). The KM analysis revealed median survival of a high prognostic score to be significantly less than that of a low prognostic score, 37.6 vs. 75.9 months, respectively (Figure 5).

### 2.7. Comparison with Normal TCGA Datasets

**Figure 6:**
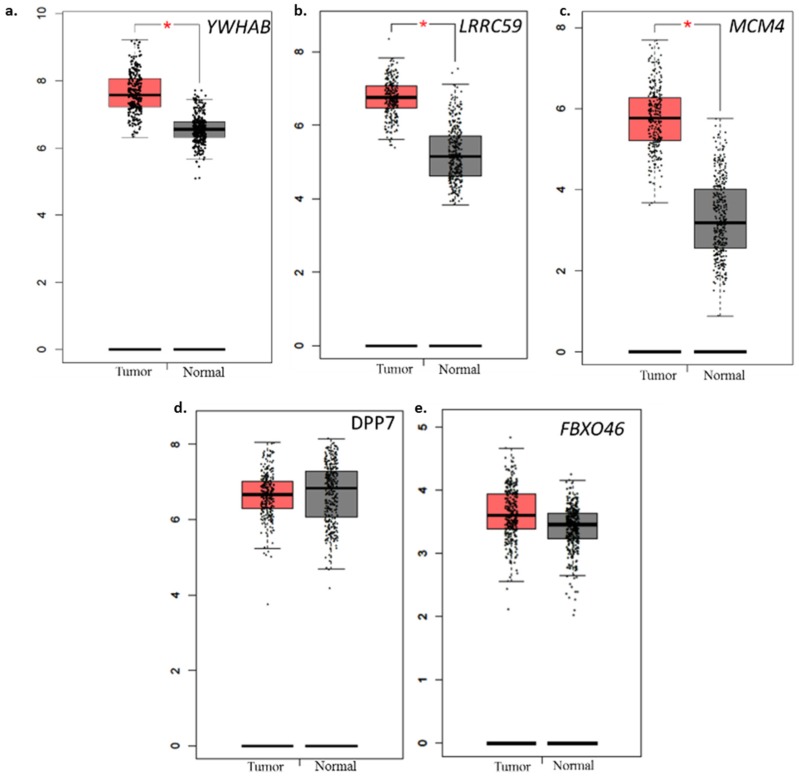
Differential expression of prognostic genes in cancerous tissue compared to normal. The expression of (**a**) *YWHAB*, (**b**) *LRRC59*, (**c**) *MCM4*, (**d**) *DPP7/2*, (**e**) *FBXO46* was assessed using normal tissue expression data from TCGA and GTEx dataset (*n* = 349) and TCGA CRC tumor dataset (*n* = 275). Higher expression of these genes except *DPP7/2* and *FBXO46* were significantly associated with tumors in CRC patients.

To further explore the variations observed in our data, the differential gene expression between normal and colon adenocarcinoma dataset was accessed through GEPIA portal. *YWHAB*, *LRRC59* and *MCM4* was significantly overexpressed in tumor tissue (*p* < 0.05) (Figure 6). *FBXO46* showed slightly higher expression in cancer tumors but did not reach statistical significance. *DPP7/2* was found to be lower in tumor tissue.

### 2.8. Biological Features of Significant Genes Found in This Panel

The functional role of the significant genes in this panel are presented in Table 6. *YWHAB* plays a role in signal transduction and cell cycle. *MCM4* plays an essential role in DNA replication. *DPP7/2* is associated with apoptosis. *FBXO46* plays a role in cancer biogenesis and *LRRC59* promotes angiogenesis and can fuel tumor growth.

### 2.9. Correlation Cluster of Expressed Genes

The Correlation cluster analysis was performed on Nanostring expression data acquired from clinical FFPE tissue blocks. All the 17 genes from the panel were clustered on the basis of spearman correlation (Figure S2). *FBXO46* correlated positively with *YWHAB* (0.95, *p* < 0.0001) and *DPP7/2* (0.90, *p* < 0.0001). *DPP7/2* showed negative correlation with *LRRC59* (−0.49, *p* < 0.0001) and *PCMT1* (−0.50, *p* < 0.0001).

## 3. Discussion

CRC is the third deadliest cancer in the United States. It is essential to develop and validate new gene expression-based prognostic markers that can predict clinical outcomes more effectively. The present study was conducted with two goals: first, as a single biomarker is not scalable to larger population, we set out to generate a robust composite four gene prognostic score to predict survival status in CRC patients; and second, to further validate some of the massive amount of data has been generated through TCGA and other databases. Additionally, in this study, African-American and Caucasian patient’s sample along with other parameters provided an opportunity to explore variations in gene expression based on various clinic-pathological characteristics. There was an effort to identify new prognostic genes as the African-American population has higher rate of incidence and mortality due to CRC [29]. This study analyzed in silico RNA seq data from TCGA and built on it to develop and experimentally validate a prognostic model through Nanostring analysis. In addition to screening of CRC prognostic genes from The Protein Atlas, genes with prognostic utility in 6 or more cancers were also included. The rationale of this top-down selection was to check the clinical significance of these genes in CRC patients. As these genes are aberrant in multiple cancers, they might be playing an important role in CRC tumorigenesis and could yield promising prognostic information. The four-gene signature, *YWHAB*, *MCM4, FBXO46* and *DPP7/2* (HR 5.39, 95% CI: 2.19–15.26, *p* < 0.001 *), was developed after multivariate Cox proportional hazard regression on the mRNA expression data from Nanostring analysis. In univariate Cox regression analysis, only stage showed prognostic correlation with overall survival (HR 2.9, 95% CI: 1.39–6.36, *p* < 0.001 *). In multivariate cox regression model, the stage and prognostic score maintained strong correlation with overall survival. Interestingly, alcohol consumption and tobacco consumption showed inverse correlation with overall survival. All the genes in the final prognostic model play a role in cancer growth and progression. Unexpectedly, 3 of the 4 genes are from the gene list with prognostic value in > 6 cancers (*YWHAB*, *MCM4, FBXO46*). This hints at the previously unidentified role of these genes in CRC tumorigenesis and prognosis. One of the genes, *YWHAB*, is included in metastatic-prone 54 gene signature for colorectal cancer [22]. Genetic alterations in *YWHAB* are observed in large scale integrated genomic analysis in multiple cancers [23]. Further, it has been revealed that B-cell translocation gene (*BTG3*) knockdown is related to over-expression of multiple genes including *YWHAB* in colorectal cancer [30]. As *YWHAB* is involved in multiple signaling pathways inside the cell, it might act downstream of genes like *BTG3* in CRC carcinogenesis [30]. In another proteomics study, the differential expression of *YWHAB* was quantified using a comparative MALTI/TOF analysis in response to anti-tumor response of retinoic acids [31]. Although *LRRC59* was not part of the 4 gene prognostic score it showed higher expression in tumor tissue and was found to be associated with stage and overall survival (Table S4). *LRRC59* is involved in chromosomal rearrangement in multiple cancers [28]. *LRRC59* binds to Fibroblast growth factor 1 (FGF1) and imports it into the nucleus [24]. FGFs are known to promote tumor angiogenesis by their synergistic action with Vascular Endothelial Growth Factor (VEGF) [25]. *LRRC59* is associated with a significantly poorer prognosis in breast cancer [32]. Additionally, *LRRC59* has been shown to transport CIP2A (cancer inhibitor of PP2A) into the nucleus, disrupting mitotic checkpoints and deregulating the cell cycle in prostate cancer cells [33]. The minichromosomal maintenance (MCM) proteins play an essential role in DNA replication [34]. The dysregulation of MCM proteins has been linked with cancer and has been a promising prognostic marker, especially in esophageal adenocarcinoma and pancreatic lesions [26]. *DPP7/2* encodes aminopeptidases which are expressed in both quiescent lymphocytes and fibroblasts, maintaining a G_0_ state and inhibiting apoptosis. As p53 regulates the *DPP7/2* promoter, reduced expression is associated with cell cycle deregulation, as well as induction of c-Myc [35]. Interestingly, the inhibition of DPP7/2 induces apoptosis in resting lymphocytes but not activated lymphocytes. To this end, DPP7/2 driven apoptosis has been shown to be reliable prognostic factor in chronic lymphocytic leukemia (CLL), as CLL B-cells sensitive to DPP7/2 inhibition are in G_0_, while resistant CLL B-cells are partially activated [27]. *FBXO46* has not been as thoroughly characterized as the other prognostic genes, but it has been found to be dysregulated in cancer and plays a role in biogenesis of cancer [36].

Among 17 genes that were included in this panel, the expression of *YWHAB*, *MCM4*, *LRRC59* and *FBXO46* was found to be elevated in tumor tissue compared to normal. Although non-significant, *DPP7/2* was expressed at slightly higher levels in normal tissue. This may be due to expression being limited to only a subset of quiescent lymphocytes and fibroblasts. Patients with lower expression of *DPP7/2* had poorer overall survival in our study. In correlation analysis, *FBXO46* was found to be highly correlated with *YWHAB* (Pearson χ^2^ test, *p* < 0.0001). In combination, they might play a significant role in CRC tumorigenesis. In another significant correlation, *DPP7/2* showed negative correlation with *LRRC59* (Pearson χ^2^ test, *p* < 0.0001) and *PCMT1* (Pearson χ^2^ test, *p* < 0.0001). As the expression of *DPP7/2* is downregulated in CRC tumor tissues, it shows inverse correlation with *PCMT1*, which has been shown to express at higher amounts in bladder cancer [37].

The prognostic score generated in this study was also evaluated for stage-specific prognostic significance. Identification of low risk patients in stage II and III is critical as several studies have found that only surgery is sufficient to cure most of the patients and chemotherapy was beneficial only for only a subset of patients [38]. If a novel prognostic method is developed, these low risk patients could be spared from toxic effects and numerous sequelae of chemotherapy. Several gene expression signature-based tests are currently being validated in larger cohorts, but multiple new signatures are continuously being reported [39,40,41]. There are several studies which have identified single gene like *PDL-1*, *Layilin* and *Apolipoprotein E* with prognostic significance in colorectal cancer [42,43,44]. There are several multiple gene signatures also that have been reported to divide patients on the basis of overall survival [45,46]. In this study, the utilization of a unique approach to include genes with prognostic significance in > 6 cancers added novelty to the 17 gene panel. These novel genes can assist in a more accurate prognosis of patients, especially stage II and stage III, which might not be as accurately defined through other gene panels. While databases such as Oncomine can be valuable tools, expression values might differ in tumor tissues for this prognostic gene signature, most likely due to the lack of survival data and clinical information. Our study attempts to find a consensus prognostic score after utilizing TPA, cBioportal, Nanostring and GEO datasets. To maximize the clinical impact in a specific stage, a recent study utilized a Random Forest analysis to identify 8 gene-signature for risk stratification in stage I of AJCC [47]. Our prognostic signature significantly differentiated patients based on overall survival and maintained significance for stage II stage III patients, which are prognostically difficult to differentiate. This stage specific risk score generation lends specificity to prognostic scores, increasing accuracy in the clinical setting. Future validation of these genes in larger cohorts including colorectal cancer specific functional and regulatory roles remains to be elucidated.

## 4. Materials and Methods

### 4.1. Data Source and Generation of 17-Gene Panel

**Figure 7:**
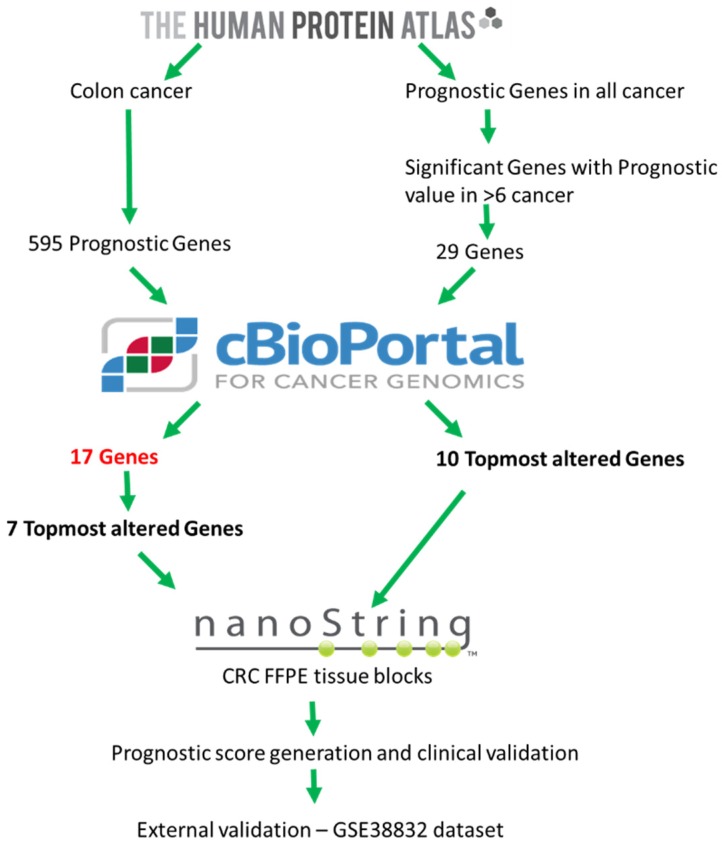
A flowchart depicting gene extraction methodology for generation of 17 gene panel.

**Figure 8:**
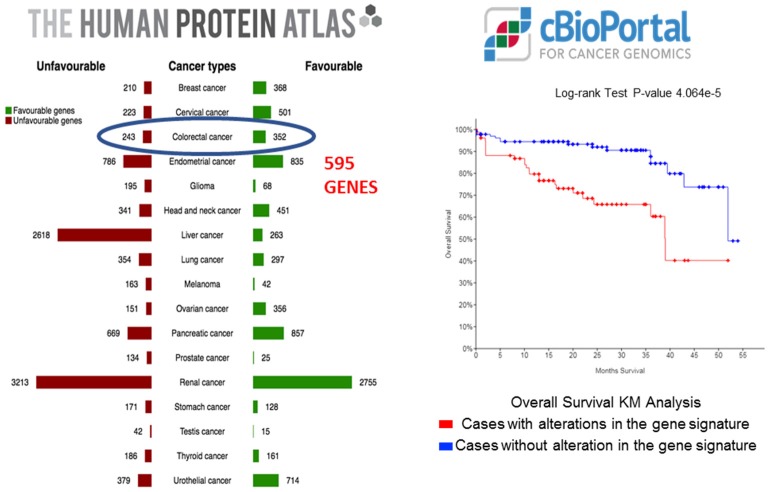
The 595 candidate gene-set was extracted from The Protein Atlas. The gene expression of 7 top-most altered genes (*PI4K2B*, *PBXIP1*, *CHEK1*, *DLAT*, *FAM50A*, *KDM4B*, *DPP7/2*) significantly differentiated patients on the basis of overall survival in cBioportal, with perturbations in 37% of CRC patients (*n* = 222).

**Figure 9:**
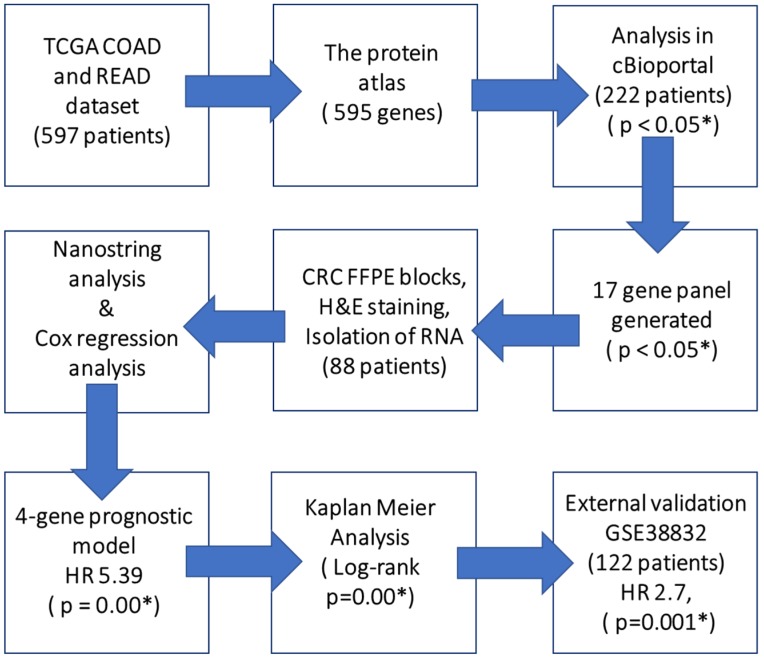
A flowchart depicting the process used to identify, generate and validate the prognostic signature in colorectal cancer.

The exploratory TCGA cohort consisted of 597 CRC patients. The extraction of 595 candidate genes for CRC was performed through The Human Protein Atlas (TPA) (https://www.proteinatlas.org) (Figure 7 and Figure 8). The gene list was downloaded in .tsv format and was stratified on the basis of the individual gene’s significance in OS prognosis of CRC. Next, these genes were screened for their combined prognostic significance in cBioportal (http://www.cbioportal.org) (Tables S1 and S2). The cBioportal is an online database with mRNA expression data derived on the Agilent microarray platform with a colon adenocarcinoma cohort of 222 samples. Genes were queried with an mRNA expression z-score threshold value of ± 2.0. Genes not reaching significant variable expression from the 595 candidate genes were removed through backward deletion, leaving 7 significantly altered genes in cBioportal (*PI4K2B, PBXIP1, CHEK1, DLAT, FAM50A, KDM4B, DPP7/2*) (*p* < 0.0001). In combination, the expression of these genes significantly differentiated CRC patients on the basis of overall survival (Figure 8). As Multiple platforms like TPA and cBiportal helps in discovery and screening of potential candidate prognostic gene before it’s validation on clinical samples. To expand the gene panel and to discover new prognostic genes, a novel strategy was utilized to include genes with aberrant expression in multiple cancers. For this a total of > 10,000 genes with prognostic significance in 17 cancers were downloaded from TPA database. Of these twenty-nine genes showed significant variable expression in 6 or more diverse types of cancer. These genes were queried in cBioportal for their significance in CRC, and the top 10 altered genes on the basis of percent altered samples, were added to the panel (*YWHAB*, *DSG2*, *PCMT1*, *MCM4*, *AGFG1*, *E2F1*, *LRRC59*, *SLAMF6*, *FBXO46*, *ITGA5*) (Tables S2 and S3). In the initial screening of aforementioned 7 genes and 10 genes, it was made sure that individual gene was altered in >5% of cBioportal screening dataset. The role of these genes in CRC prognosis was tested using clinical and external dataset. For external validation, human expression profile dataset of an independent CRC study (GSE38832, *n* = 122) was downloaded from Gene Expression Omnibus (GEO) database (https://www.ncbi.nlm.nih.gov/geo). The GSE38832 study was performed using an Affymetrix Human Genome U133 Plus 2.0 Array. The downloaded data was further curated for all the relevant clinical and follow-up data features. The flowchart of the entire study is depicted in (Figure 9).

### 4.2. Patient Characteristics

For internal validation, Formalin Fixed Paraffin Embedded (FFPE) blocks were accessed from pathology archives at the Medical College of Georgia at Augusta University, Augusta, GA 30912, USA. Under an IRB approved protocol (HAC # 611298), CRC patients with 5 years’ follow-up were included in this study. A total of 88 patients from all the 4 stages fit in our inclusion criteria on the basis of survival duration after diagnosis. A total of 26 patients were administered chemotherapy after surgery and 62 patients did not receive any chemotherapy. No Informed consent from the patients was required as this was a retrospective study on de-identified FFPE samples. The patients were stratified on the basis of overall survival in two groups, with higher (patient that survived >3 years) and lower survival (patient that survived <1 year) along with American Joint Committee on Cancer (AJCC) staging system (I to IV), grade, gender, age, distant metastasis, location and vital status. Only histologically confirmed cancer patients were included in this study. The samples with insufficient documentation, lack of tumor tissue in blocks, failure of RNA isolation or highly degraded RNA were not included in this study.

### 4.3. FFPE Tissue Sectioning and H&E Staining

FFPE blocks were used to produce fine sections for further microscopic analysis and RNA isolation. For tissues that had rich cancerous region, only five 5 μm sections were cut and for small tissues twenty sections were cut. H&E staining was performed using standard protocol and was examined for tumor-rich regions by a board-certified pathologist.

### 4.4. RNA Isolation

Total RNA was isolated through miRNEasy FFPE kit (Qiagen, Hilden, Germany) using standard protocol. The eluted RNA was quantified using Nanodrop spectrophotometer (NanoDrop ND-1000, NanoDrop Technologies, Wilmington, NC, USA).

### 4.5. Quantification of mRNA Molecules Using Nanostring Platform

To quantify mRNA expression of 17 genes, we employed multiplex, high-throughput digital quantification instrument by Nanostring (NanoString Technologies Inc., Seattle, WA, USA). Additionally, 6 control genes were also quantified for normalization of gene expression. A total of 300 ng of total RNA was used as an input for this analysis. Nanostring and its nCounter PlexSet technology is a digital quantification system, which quantifies RNA molecules using a target specific oligonucleotide probe pairs in a highly specific manner. PlexSet contains uniquely coded fluorescent barcodes that are linked to reporter tags and a biotinylated universal capture tag. The reporter tags emit a unique signature fluorescence that is individually resolved and counted during data capture and analysis. On the other hand, the universal capture tag anchors specific RNA molecules to streptavidin-coated lane on the nCounter instrument [48]. The Nanostring assay was performed as per the manufacturer’s instructions. The data collection that involves detection, resolution and quantification of individual florescent barcodes was performed later on a separate instrument, nCounter Digital Analyzer (DA). The fields of view (FOV) setting for DA was set at 280 FOV, as previously noted [49].

### 4.6. mRNA Expression Data Normalization

The raw gene expression counts were processed and normalized according to the manufacturer’s recommendations (NanoString Technologies Inc., Seattle, WA, USA). The geometric mean of the negative and positive control was used to normalize the data. The second normalization was later performed using 6 internal control genes (*ABCF1*, *GUSB*, *HPRT1*, *LDHA*, *POLR1B*, *RPLO*). The normalizations were performed using the nCounter software (NanoString Technologies Inc., Seattle, WA, USA).

### 4.7. Correlation Analysis and Gene Expression Comparison with Normal Tissue

For correlation among the genes, cluster analysis of 17 genes was performed on the basis of Spearman correlation coefficient. For normal and tumor tissue expression comparison, Gene Expression Profiling Integrative Analysis (GEPIA) database (http://gepia.cancer-pku.cn) was utilized. In GEPIA, the COAD tumor (*n* = 275) dataset was compared against combined gene expression data of normal tissues from TCGA and Genotype-Tissue Expression (GTEx) data (*n* = 349). In GEPIA, standard parameters with Log_2_FC cutoff was set at 1 and *p*-value cut-off at 0.01 were used.

### 4.8. Construction and Validation of a 4 Gene Prognostic Model

The prognostic score was generated using the Cox proportion regression coefficient for each gene. For every patient the prognostic score was calculated by multiplying the expression value of a gene with its corresponding Cox proportion regression coefficient (Prognostic score = Σ Cox regression coefficient of Gene_i_ * expression value of gene Gene_i_). Separate coefficients were calculated for both internal and external datasets. The resulting prognostic score based on these coefficients was used to divide patients into categorical variables, i.e., high score and low score groups based on median cut-off threshold. This categorical variables were utilized to differentiate patients in stage II and stage III from the internal and external datasets. The KM Analysis was performed to assess the utility of this model to differentiate these groups.

### 4.9. Statistical Analysis

The continuous variables in this study including Nanostring expression counts are shown as the mean ± SE. The median of the normalized counts was taken to divide patients into two groups—individuals with higher expression and with lower expression. The relationship between gene expression of these groups were compared with the categorical clinic-pathological parameters using Pearson χ^2^ test. The univariate and multivariate analysis of different genes was performed using Cox proportion hazard regression method. The Hazard ratio and 95% confidence interval values were also derived from Cox proportion hazard model. Kaplan-Meier method was used to analyze survival and log-rank test was used to calculate the differences in their distribution. The calculations of *p*-values were two-sided, and *p* < 0.05 was defined as statistically significant. Additionally, ROC (Receiver operating characteristic) analysis was performed for 4 gene signature on both external and clinical datasets. The statistical analyses were conducted using JMP-Pro (version 14.0.0, SAS Institute, Cary, USA) and GraphPad Prism (version 8 GraphPad Software, La Jolla California USA).

## 5. Conclusions

In summary, our study developed a novel four gene prognostic model which has been used to predict clinical outcomes in CRC patients. Our approach to first identify risk genes from TCGA datasets and validate experimentally can be equally insightful in other cancers. There is additional research required to assess the functional role of these genes in colorectal tumors. We are in the process of validating this study on a larger cohort and independent datasets. The efforts to develop similar gene signatures promises to equip clinicians with better information to adopt novel personalized interventions for higher risk patients.

## Figures and Tables

**Table 1:** The 17-gene panel included in this study along-with its expression variation in tumor and normal tissues as accessed from GEPIA portal.

S.No	Gene Symbol	Entrez Gene ID	Cytoband	Gene Title	Median Gene Expression, TPM (Transcripts per million)	
					Tumor (*n* = 275)	Normal (*n* = 349)
**Genes selected from TPA and cBioportal**						
1	PI4K2B	55300	4p15.2	Phosphatidylinositol 4-kinase type 2 beta	0.05	0
2	PBXIP1	57326	1q21.3	Pre-B-cell leukemia homeobox interacting protein 1	31.3	75.75
3	CHEK1	1111	11q24.2	Checkpoint kinase 1	18.12	2.5
4	DLAT	1737	11q23.1	Dihydrolipoamide S-acetyltransferase	23.51	15.09
5	FAM50A	9130	Xq28	Family with sequence similarity 50, member A	66.45	62.17
6	KDM4B	23030	19p13.3	Lysine (K)-specific demethylase 4B	9.39	12.39
7	DPP7/2	29952	9q34.3	Dipeptidyl-peptidase 7	100.63	113.3
**Genes selected from prognostic significance in multiple cancers**						
8	YWHAB	7529	20q13.1	Tryptophan 5-monooxygenase activation protein, beta	190.47	93.05
9	DSG2	1829	18q12.1	Desmoglein 2	76.26	3.67
10	PCMT1	5110	6q25.1	Protein-L-isoaspartate (D-aspartate) O-methyltransferase	48.97	38.82
11	MCM4	4173	8q11.2	Minichromosome maintenance complex component 4	53.46	8.09
12	AGFG1	3267	2q36.3	ArfGAP with FG repeats 1	45.46	24.07
13	E2F1	1869	20q11.2	E2F transcription factor 1	13.51	2.13
14	LRRC59	55379	17q21.33	Leucine rich repeat containing 59	107.08	34.6
15	SLAMF6	114836	1q23.2	SLAM family member 6	1.38	0.82
16	FBXO46	23403	19q13.3	F-box protein 46	11.1	9.98
17	ITGA5	3678	12q11-q13	Integrin alpha 5	21.76	191.95

**Table 2:** Demographic and clinical information of colorectal cancer patients included in this study.

Clinical Parameters	No. of Patients	Percentage of Patients (%)
**Age**		
<68 y	27	30.68
>68 y	61	69.32
**Gender**		
Male	37	42.05
Female	51	57.95
**Stage - AJCC**		
I	14	15.91
II	30	34.09
III	26	29.55
IV	18	20.45
**Grade**		
I - Well differentiated	18	20.45
II: Intermediate differentiated	40	45.45
III: Poorly differentiated	23	26.14
IV: Undifferentiated	7	7.95
**Distant Metastasis**		
Yes	33	37.5
No	54	61.36
**Vital Status**		
Dead	57	64.77
Alive	31	35.23
**Ethnicity**		
Caucasian	47	53.41
African-American	38	43.18
**Alcohol Use**		
No Usage	67	76.14
Users	20	22.73
**Tobacco Use**		
No	56	63.64
Yes	32	36.36
**Chemotherapy after surgery**		
Administered	26	29.54
Not administered	62	70.45
**Family History**		
No	41	46.59
Yes	35	39.77
**Months survival (median)**		
Dead	11.8 months	
Alive	54.1 months	

**Table 3:** Univariate Cox regression analysis of the genes included in this panel.

Gene	Univariate		
	Hazard Ratio	95% CI	*p*-Value
*CHEK1*	0.66	0.31–1.37	0.26
*DLAT*	0.7	0.33–1.48	0.35
*DPP7/2*	0.38	0.14–0.89	0.02
*FAM50A*	0.5	0.19–1.45	0.2
*KDMB*	0.72	0.31–1.57	0.41
*PBXIP1*	1.14	0.51–2.66	0.74
*PI4K2B*	1.09	0.52–2.38	0.8
*DSG*	1.3	0.66–2.93	0.39
*E2F*	1.77	0.79–4.05	0.15
*MCM4*	2.69	1.19–6.35	0.01
*PCMT1*	0.66	0.30–1.41	0.28
*YWHAB*	3.76	1.58–9.66	0.001
*AGFG1*	0.8	0.39–1.82	0.65
*FBXO46*	1.4	0.65–2.94	0.37
*ITGA5*	0.71	0.34–1.48	0.37
*LRRC59*	2.32	1.09–5.35	0.02
*SLAMF6*	1	0.50–2.45	0.81

**Table 4:** Univariate Cox regression analysis of prognostic score and other clinicopathological variables.

Variable	Univariate		
	Hazard Ratio	95% CI	*p*-Value
Prognostic score (composite *DPP7/2*, *YWHAB*, *MCM4* and *FBXO46*)	5.39	2.19–15.26	<0.001 *
Age (>68, <68 years)	0.78	0.37–1.66	0.51
Gender (Male, Female)	0.95	0.45–2.06	0.89
Stage (III + IV, I + II)	2.9	1.39–6.36	<0.001 *
Grade (III, I + II)	1.79	0.73–5.3	0.2
Ethnicity (African-American, Caucasian)	0.9	0.41–2.07	0.81
Alcohol consumption (Yes, No)	0.6	0.26–1.55	0.27
Tobacco smoking (Yes, No)	0.58	0.25–1.23	0.16

**Table 5:** Multivariate Cox regression analysis of prognostic score in combination with other clinicopathological variables.

Variable	Multivariate		
	Hazard Ratio	95% CI	*p*-Value
Prognostic score (composite *DPP7/2*, *YWHAB*, *MCM4* and *FBXO46*)	3.42	1.71–7.94	<0.001 *
Age (>68, <68 years)	1.05	0.35–3.19	0.92
Gender (Male, Female)	2.34	0.62–9.37	0.20
Stage (III + IV, I + II)	4.56	1.33–19.15	0.01 *
Grade (III, I + II)	0.19	0.02–1.18	0.07
Ethnicity (African-American, Caucasian)	2.89	0.69–12.47	0.14
Alcohol consumption (Yes, No)	7.38	1.58–38.14	0.01 *
Tobacco smoking (Yes, No)	0.08	0.01–0.31	0.01 *

**Table 6:** Functional relevance of genes that were significantly associated with OS in CRC patients.

Gene	Function and Role in Cancer	References
*YWHAB*	Signal transduction and cell cycle, genetically altered in multiple cancers	[22,23]
*MCM4*	Essential role in DNA replication, dysregulation found in several cancers.	[24,25]
*DPP7/2*	Inhibition of DPP7/2 has been linked with apoptosis through c-Myc and p53 related pathways	[26]
*FBXO46*	Deregulated cell cycle, cancer biogenesis	[27]
*LRRC59*	Essential for nuclear import of Fibroblast growth factor 1, FGF promotes angiogenesis with VEGF	[24,28]

## Supplementary Materials

Supplementary materials can be found at https://www.mdpi.com/1422-0067/20/15/3818/s1.
