# Supplementary material for: Identification and Clinical Validation of a Novel 4 Gene-Signature with Prognostic Utility in Colorectal Cancer
Source: Int J Mol Sci. 2019 Aug 5;20(15):3818. doi: 10.3390/ijms20153818 (PMC6696416; doi:10.3390/ijms20153818)
Supplement: Supplementary file 1 [file ijms-20-03818-s001.zip › ijms-559996-SI.pdf]

Supplementary materials

Table S1. Top-most genes from initial 595 genes extracted from TPA's CRC prognostic genes analyzed in cBioportal.

Gene Symbol	Number of Samples Altered	Percent Samples Altered	Log-rank *p*-value
PI4K2B	22	10.00%	0.011
PBXIP1	18	8.20%	< 0.001
CHEK1	17	7.70%	0.052
DLAT	17	7.70%	0.154
FAM50A	15	6.80%	0.956
KDM4B	14	6.40%	0.031
DPP7	11	5.00%	0.058
WNT5A	10	4.50%	< 0.001
HSPA8	9	4.10%	0.111
SORT1	9	4.10%	0.685
RBM3	9	4.10%	0.082
PHF1	9	4.10%	0.143
DAPK1	8	3.60%	0.0412
REP15	8	3.60%	0.032
DDX46	5	2.30%	0.435
LRCH4	0	0.00%	
DEFA5	0	0.00%	

Table S2. Distribution of 29 genes extracted from TPA's prognostic genes with perturbations in > 6 cancers as analyzed through cBioportal on TCGA-COAD CRC dataset. These top 10 altered genes were selected and included in the panel.

Gene	Num Samples Altered	Percent Samples Altered
YWHAB	86	39.10%
DSG2	47	21.40%
PCMT1	24	10.90%
MCM4	19	8.60%
AGFG1	18	8.20%
E2F1	18	8.20%
LRRC59	16	7.30%
SLAMF6	15	6.80%
FBXO46	13	5.90%
ITGA5	12	5.50%
STC1	12	5.50%
C3ORF62	11	5.00%
CD3D	11	5.00%
NCAPG2	11	5.00%
SPOCK1	11	5.00%
ANXA5	10	4.50%
CD3E	10	4.50%
TUBA1C	10	4.50%
ZNF101	10	4.50%
ZNF266	10	4.50%
ANXA2	9	4.10%
CD2	9	4.10%
CD5	9	4.10%
P4HA1	8	3.60%
SCRN1	8	3.60%
CSAD	7	3.20%
S100A10	6	2.70%
SERPINE1	4	1.80%
CKS1B	0	0.00%

Table S3. The prognostic roles of 10 genes included in 17-gene panel in other cancers.

Gene	Protein Location	Prognostic Value in Cancers
YWHAB	Cytosol	Liver cancer:1.91e-6 , Renal cancer:2.52e-5 , Endometrial cancer:5.46e-4 , Lung cancer:6.01e-4 , Head and neck cancer:6.14e-4 , Breast cancer:9.18e-4
DSG2	Plasma membrane, Cell Junctions	Renal cancer:3.14e-10 , Pancreatic cancer:4.94e-5 , Lung cancer:2.43e-4 , Head and neck cancer:5.63e-4 , Colorectal cancer:7.81e-4 , Cervical cancer:8.22e-4
PCMT1	Cytosol	Breast cancer:4.02e-7 , Head and neck cancer:1.88e-4 , Liver cancer:2.58e-4 , Endometrial cancer:4.27e-4 , Urothelial cancer:6.38e-4 , Cervical cancer:9.58e-4
MCM4	Nucleoplasm	Endometrial cancer:2.84e-5 , Melanoma:1.15e-4 , Liver cancer:1.23e-4 , Colorectal cancer:2.19e-4 , Pancreatic cancer:2.38e-4 , Renal cancer:2.61e-4
E2F1	Nucleoplasm, Centrosome	Cervical cancer:9.22e-6 , Liver cancer:3.83e-5 , Endometrial cancer:4.33e-5 , Renal cancer:8.99e-5 , Thyroid cancer:1.62e-4 , Pancreatic cancer:4.18e-4
AGFG1	Vesicles	Liver cancer:7.31e-6 , Ovarian cancer:3.43e-5 , Lung cancer:1.85e-4 , Renal cancer:6.92e-4 , Cervical cancer:7.13e-4 , Glioma:7.89e-4
LRRC59	Endoplasmic reticulum	Renal cancer:1.19e-6 , Liver cancer:3.12e-6 , Head and neck cancer:1.00e-5 , Urothelial cancer:1.90e-5 , Colorectal cancer:2.60e-4 , Pancreatic cancer:6.87e-4
SLAMF6	plasma membrane	Renal cancer:2.09e-6 , Head and neck cancer:7.34e-5 , Melanoma:1.25e-4 , Cervical cancer:2.78e-4 , Breast cancer:9.42e-4 , Endometrial cancer:9.53e-4
FBXO46	Nucleus, Vesicles, Cytosol	Urothelial cancer:5.09e-6 , Endometrial cancer:2.13e-5 , Liver cancer:1.11e-4 , Renal cancer:5.33e-4 , Head and neck cancer:5.44e-4 , Pancreatic cancer:8.11e-4

ITGA5	plasma membrane	Renal cancer:1.32e-8 , Cervical cancer:4.07e-6 , Liver cancer:2.23e-5 , Lung cancer:2.44e-4 , Head and neck cancer:2.91e-4 , Colorectal cancer:8.43e-4 , Thyroid cancer:8.89e-4

Table S4. Chi-square analysis of clinico-pathological variables and gene expression based on median cut-off value.

	DPP7/2			*MCM4*			*FBXO46*		
**Variable (n)**	**Lower**	**Higher**	***p*-value**	**Lower**	**Higher**	***p*-value**	**Lower**	**Higher**	***p*-value**
**Age**			0.1			0.48			0.24
<68 y (27)	17	10		15	12		16	11	
>68 y (61)	27	34		29	32		28	33	
**Gender**			0.82			0.13			0.13
Male (37)	19	18		15	22		22	15	
Female (51)	25	26		29	22		22	29	
**Stage**			0.66			0.2			0.66
I+II (44)	21	23		25	19		23	21	
III + IV (44)	23	21		19	25		21	23	
**Grade**			1			0.8			0.36
I-II (58)	29	29		31	27		27	31	
Poor-Undiff. (30)	15	15		13	17		17	13	
**Ethnicity**			0.43			0.00*			0.43
African-american (38)	21	17		13	25		17	21	
Caucasian (47)	22	25		30	17		25	22	
**Alcohol**			0.14			0.56			0.14
Alcohol used (20)	13	17		9	11		13	7	
No alcohol used (67)	31	36		35	32		31	36	

**Tobacco**			0.65			0.65			0.37
No (56)	27	29		29	27		26	30	
Yes (32)	17	15		15	17		18	14	
**Family History**			0.48			0.98			0.81
History of cancer (35)	19	16		18	17		18	17	
No history of cancer (41)	19	22		21	20		20	21	
**Vital Status**			0.26			0.82			0.26
Dead (57)	18	13		16	15		18	13	
Alive (31)	26	31		28	29		26	31	

	YWHAB			*LRRC59*		
**Variable (n)**	**Lower**	**Higher**	***p*-value**	**Lower**	**Higher**	***p*-value**
**Age**			0.81			0.24
<68 y (27)	14	13		11	16	
>68 y (61)	30	31		33	28	
**Gender**			0.82			0.28
Male (37)	18	19		16	21	
Female (51)	26	25		28	23	
**Stage**			0.39			0.01*
I+II (44)	24	20		28	16	
III + IV (44)	20	24		16	28	
**Grade**			0.17			1
I-II (58)	26	32		29	29	
Poor-Undiff. (30)	18	12		15	15	

**Ethnicity**			0.56			0.06
African-american (38)	21	17		15	23	
Caucasian (47)	23	24		28	19	
**Alcohol**			0.95			0.33
Alcohol used (20)	10	10		8	12	
No alcohol used (67)	33	34		35	32	
**Tobacco**			0.37			0.65
No (56)	26	30		29	27	
Yes (32)	18	14		15	17	
**Family History**			0.63			0.68
History of cancer (35)	16	19		17	18	
No history of cancer (41)	21	20		18	23	
**Vital Status**			0.82			0.01*
Dead (57)	16	15		10	21	
Alive (31)	28	29		34	23	

Table S5. Multivariate Cox proportional prognostic analysis of clinical dataset in this study.

**Variable**	**Multivariate**		
	**Hazard Ratio**	**95% CI**	**p-value**
Prognostic score	2.6	1.44-5.10	<0.001*
Age (>68, <68 years)	1.35	0.52-3.51	0.52
Gender (Male, Female)			
Stage (III+IV, I+II)	3.24	1.32-8.63	0.009*
Grade (III, I+II)			
Ethnicity (African-American, Caucasian)	2.46	0.92-6.75	0.0121*

Alcohol (Yes, No) consumption	2.6	0.87-7.65	0.081
Tobacco smoking (Yes, No)			

Table S6. Univariate and Multivariate Cox proportional prognostic analysis of external dataset used in this study.

**Variable**	**Hazard Ratio**	**95% CI**	**p-value**
**Univariate Analysis**			
DPP7/2	0.45	0.29-0.69	0.0003
MCM4	3.37	2.19-5.23	<0.001
YWHAB	1.71	1.12-2.61	0.012
FBXO46	2.02	1.10-3.69	0.49
Stage (III+IV, I+II)	1.48	0.97-2.27	0.06
**Prognostic score (composite DPP7/2, YWHAB, MCM4 and FBXO46)**	**2.7**	**1.99-3.73**	**<0.001**
**Multivariate Analysis**			
Prognostic score (composite *DPP7/2*, *YWHAB*, *MCM4* and *FBXO46*)	3.67	2.32-5.87	<0.001
Stage (III+IV, I+II)	1.49	0.98-2.30	0.06

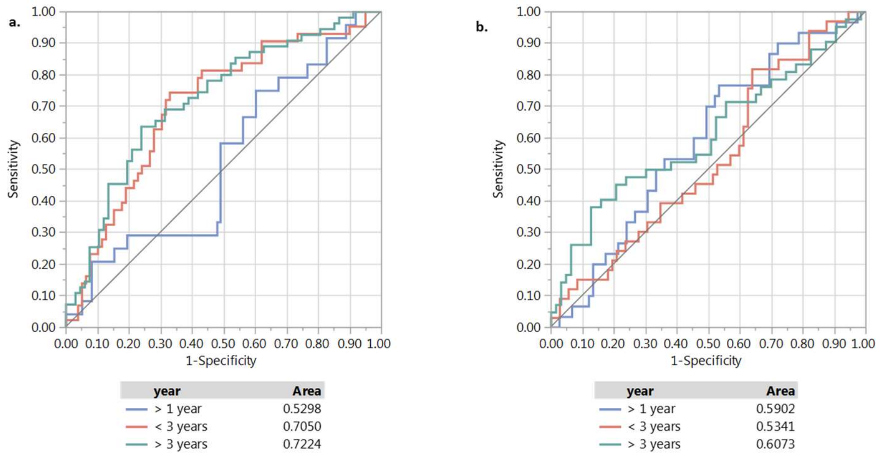

Figure S1. ROC analysis of prognostic gene signature (**a**) In external dataset, The AUC value of survival at > 1 year, < 3 years and > 3 years is 0.529 (p = 0.014), 0.705 (p = 0.002) and 0.722 (p < 0.001) respectively. (**b**) In Internal dataset, The AUC value of survival at > 1 year, < 3 years and > 3 years is 0.590 (p = 0.44), 0.534 (p = 0.16) and 0.607 (p = 0.64) respectively.

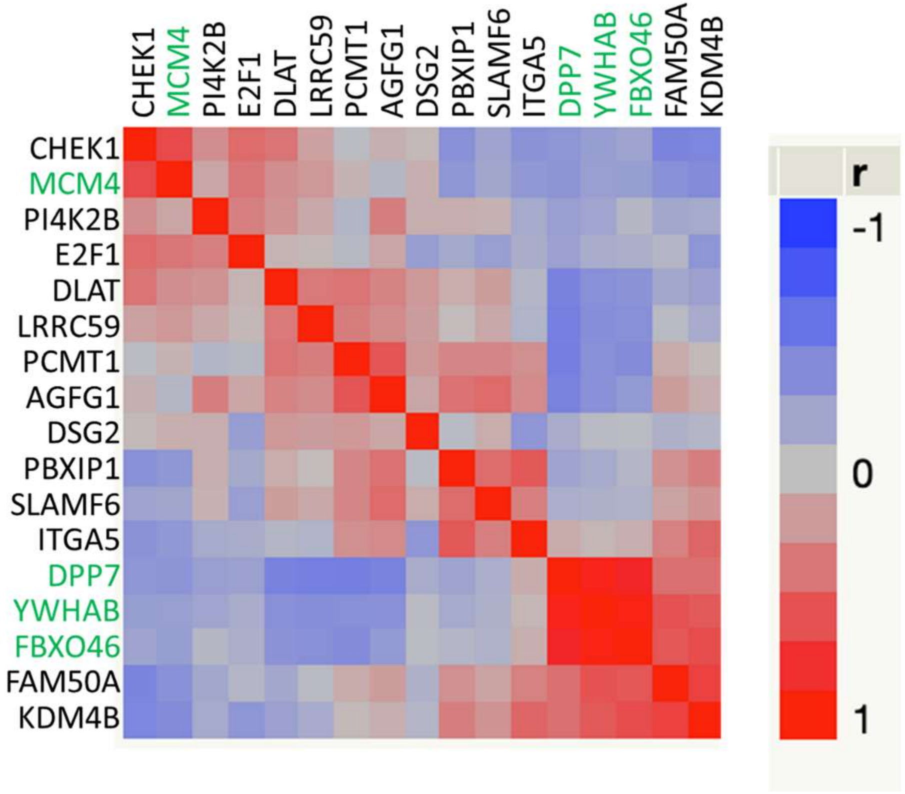

Figure S2. Cluster analysis of 17 genes included in this panel based on the Spearman correlation coefficient. The red color in the heat map indicates a positive correlation and blue indicates negative correlation. The genes included in the final 4-gene signature are colored green.
